# The immune system of preterm infants: an overview

**DOI:** 10.3389/fimmu.2026.1810170

**Published:** 2026-06-05

**Authors:** Mirjam J. Esser, Sanne J. C. M. Claassen, Melania P. Ebrahimi, Stan Berkers, Tim G. A. M. Wolfs, Magdalena A. Berkowska, Gertjan J. A. Driessen, Else M. Bijker

**Affiliations:** 1 Department of Pediatrics, Maastricht University Medical Center, MosaKids Children’s Hospital, Maastricht, Netherlands; 2 Research Institute for Oncology and Reproduction (GROW), Maastricht University, Maastricht, Netherlands; 3 Laboratory of Pediatrics, Maastricht University Medical Center, MosaKids Children’s Hospital, Maastricht, Netherlands; 4 Department of Immunology, Erasmus Medical Center (MC), University Medical Center, Rotterdam, Netherlands; 5 Maastricht-Nijmegen Translational Research Alliance for Innovative Vaccinology (TRAIN), Maastricht, Netherlands; 6 Department of Paediatrics, Oxford Vaccine Group, University of Oxford, Oxford, United Kingdom; 7 Care and Public Health Research Institute (CAPHRI), Maastricht University, Maastricht, Netherlands

**Keywords:** adaptive immunity, antibodies, B cells, immune system, innate immunity, preterm, T cells, vaccination

## Abstract

Every year, approximately 13 million infants are born preterm (<37 weeks gestation). Preterm-born infants experience disproportionately high infection-related morbidity and mortality, reflecting the immaturity of their immune system, especially early in life. This review provides an up-to-date overview of the phenotype and function of the immune system in preterm infants compared with term infants, with an emphasis on adaptive immunity. At birth, both innate and adaptive immune cells of preterm infants show phenotypic and functional immaturity. In addition, antibody levels are reduced, and immunogenicity of some vaccine components is diminished, contributing to impaired pathogen clearance and suboptimal vaccine responses. During the first year of life, rapid maturation occurs and differences with term infants become less pronounced or disappear. This review provides readers with a framework for understanding the immunologic mechanisms underlying the increased infection risk in preterm-born infants. Recognizing the all-encompassing nature of immune immaturity in preterm infants is essential for the development of integrated strategies to further improve health outcomes.

## 1 Introduction

Every year, approximately 13 million infants are born preterm (<37 weeks of gestation), and prematurity is the leading cause of neonatal death worldwide (1, 2). Infection-related morbidity and mortality are disproportionately high in this population, with a profound impact on outcomes (3). Although the increased infection risk is multifactorial, a key factor underlying this vulnerability is the immaturity of the immune system.

Immune development begins early in gestation and proceeds throughout fetal life and the postnatal period (4). After birth, the state of immunological tolerance required during fetal life shifts to effective immune responses to environmental exposure. During this period, neonates primarily depend on maternally derived antibodies and their innate immune system until they develop their own adaptive immune memory. Preterm birth interrupts normal fetal immune maturation. As a result, preterm-born infants show developmental immaturity in both innate and adaptive immunity compared with term infants (5), which may limit effective responses to pathogens and vaccines. In addition, several perinatal factors contribute to an increased infection risk, including invasive procedures, altered mucosal colonization (e.g. due to antibiotics), reduced protection from maternally derived antibodies and an immature skin barrier (6, 7).

Adaptive immunity is central to pathogen-specific protection and long-term immune defense, including the development of effective vaccine-induced responses and memory. However, the duration of adaptive immune immaturity following preterm birth and how it affects vaccination responses remains incompletely understood. This review summarizes the current knowledge of immune development in preterm infants. We describe cellular and humoral immunity, including vaccine responses, and thereby aim to provide an up-to-date overview of the immune mechanisms contributing to the vulnerability of this high-risk population.

## 2 Innate immunity

The innate immune system serves as the body’s first line of defense against infection (8). From 3–4 weeks of gestation, early innate progenitors arise in the yolk sac, with hematopoiesis subsequently shifting to the fetal liver and, by the second trimester, to the bone marrow (4, 9, 10). During gestation, neutrophils, monocytes, dendritic cells, and natural killer (NK) cells progressively increase in number and become functional (11). By term, these cells possess effective phagocytic activity and are capable of chemotaxis and cytokine production, enabling efficient pathogen recognition and clearance. In contrast, preterm neonates show immature innate immune cells with impaired effector functions, as previously reviewed (10, 12, 13). The following section highlights some of these differences in innate immune cells between preterm and term infants in more detail.

**Figure 1:**
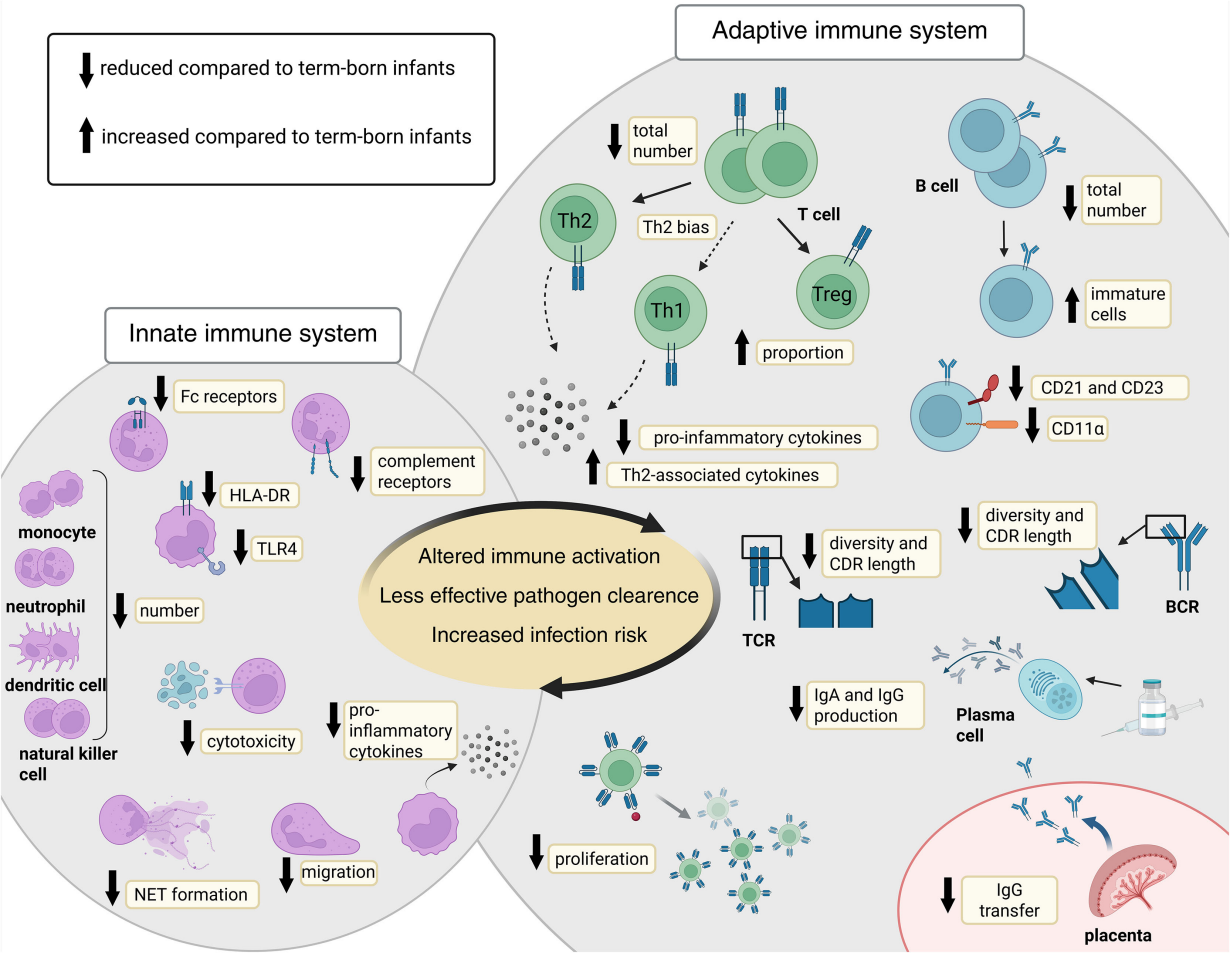
Overview of alterations in the innate and adaptive immune system of preterm-born infants. Preterm-born infants show alterations in both their innate and adaptive immune system compared with term-born infants. These include reduced cell numbers, altered subset composition, reduced expression of signaling and activation markers, and altered effector functions. Note that exposure to perinatal infection is associated with an activated immune profile, which is detailed separately in the text. Together, these impairments lead to an altered immune activation, reduced pathogen clearance, and an increased risk of infection. HLA-DR, human leukocyte antigen-DR; TLR4, toll-like receptor 4; NET, neutrophil extracellular trap, Th2, T helper 2 cell; Th1, T helper 1 cell, Treg, regulatory T cell; TCR, T-cell receptor; BCR, B-cell receptor. Created with www.biorender.com.

In preterm neonates, lower counts of neutrophils (5), monocytes (14–17), dendritic cells (15, 16), and natural killer cells (18) are described (**Figure 1**). This may contribute to the increased risk of infection in this population, as supported by findings from a study showing lower NK-cell numbers at birth in infants who later developed late-onset sepsis (18). Beyond reduced numbers, these cells show immature phenotypes and several functional impairments early after birth (19). Neutrophils from preterm infants have reduced surface expression of Fc receptors (FcγRIII) and complement receptors (CR1 and CR3), potentially contributing to diminished phagocytic activity (20, 21). While some studies indeed report reduced phagocytic activity of polymorphonuclear leukocytes (PMNs) from preterm infants against group B streptococci (22, 23), others have found an intact phagocytic capacity against *Staphylococcus aureus* and *Escherichia coli (E. coli)* (24) or a non-specific enhanced response (25). These findings suggest pathogen-specific differences in the phagocytosis function of PMNs in preterm infants. Furthermore, reduced neutrophil migration (26, 27) and neutrophil extracellular trap (NET) formation are described (28, 29). Monocytes in preterm infants have an increased proportion of phenotypically immature cells (30) and reduced expression of human leukocyte antigen-DR (HLA-DR) (31–33) and toll-like receptor 4 (TLR4) (32, 34–36), molecules involved in antigen presentation and pathogen recognition. Upon stimulation, e.g., with LPS or IFN-γ, monocytes and macrophages of preterm infants show lower upregulation of CD80 and CD86, receptors involved in T-cell activation (31, 37). Moreover, release of several cytokines, such as IL-1β, IL-8, TNF-α, IFN-γ and IL-6, upon stimulation seems impaired in monocytes from preterm infants (14, 32, 34, 38–41). Similarly, stimulated whole blood or leukocytes from preterm infants release lower levels of these pro-inflammatory cytokines (42, 43). Conversely, some studies report relatively preserved or enhanced levels of pro-inflammatory cytokines (e.g. IL-6 and IL-8) in preterm infants (44, 45), potentially related to perinatal infection (46). Elevations of these cytokines have been associated with bronchopulmonary dysplasia (BPD) and brain injury (44, 47, 48). Preterm neonates (≤32 weeks) show reduced dendritic cell activation, reflected by low CD80 expression compared to term infants (15). Consistently, lower cytokine levels in preterm infants suggest impaired stimulation or activation of Th1 cells, monocytes and dendritic cells (49). NK cells from preterm infants show reduced cytotoxicity (50, 51). Notably, in children three years of age, innate immune responses, as reflected by TLR expression and inflammatory cytokine production after stimulation, were comparable between children born preterm and term (52). Together, these examples indicate that preterm innate immune cells are reduced in number, phenotypically immature, and relatively impaired in their inflammatory responses, likely compromising pathogen recognition and clearance early in life. During the first months after birth, however, the immune system of preterm and term infants gradually converge and become phenotypically more similar (53).

## 3 Adaptive immunity

### 3.1 T cells

#### 3.1.1 T-cell numbers

##### 3.1.1.1 Total T-cell numbers at birth

T-cell development starts early in human gestation, with progenitor T cells being present in the thymus as early as 9 weeks. By week 12-13, mature T cells emerge in the thymus, and by week 24, mature T cells are present in peripheral organs such as the spleen (54). At birth, preterm-born infants have lower absolute T-cell counts compared with term-born controls (5, 18, 55–60), and this decrease is proportional to gestational age (GA) (57–59, 61) (**Figure 1**). For example, in one study, at 6–8 weeks of age, preterm infants (GA 26.5 weeks) had a median of 2816 T cells/µL (5^th^-95^th^ percentile, 1519-3837) compared to 4098 cells/µL (2409-6693) in term infants (56). Findings from newborn screening using T-cell receptor excision circles (TRECs) show that preterm infants often have lower TREC values than term infants, indicating a reduced thymic output (62–73). In line with this, preterm infants were reported to have a significantly lower proportion of CD31^+^CD4^+^ and CD31^+^CD8^+^ T cells, a subset of recent thymic emigrants containing high TREC levels (74, 75). One older study found higher absolute counts of CD2^+^ and CD4^+^ T cells in healthy preterm infants during the first week of life (76), but this finding contrasts with the broader literature and may reflect the exclusion of very low GA infants.

##### 3.1.1.2 Postnatal changes in T-cell numbers

Although longitudinal studies comparing T-cell subsets between preterm and term-born infants are scarce, they suggest that the reduction of T-cells persists beyond the neonatal period. In the first few days after birth, a burst of thymic output causes T-cell levels to rise (77), but most preterm infants continue to show lower thymic output even at term (72). Thereafter, T-cell numbers gradually increase throughout the first year of life (56, 61). In one study, preterm infants (median GA 26.5 weeks) still had significantly lower absolute total and helper T-cell numbers at 7 months of age compared to term-born controls (56). In contrast, at 3 years of age, both CD4^+^ and CD8^+^ T-cell numbers in preterm-born children (mean GA at birth 32 weeks) were comparable to those in term-born children, suggesting that normalization may occur somewhere in early childhood (52). Nevertheless, small differences in subsets may persist in very preterm-born infants. In a study of schoolchildren (median age 8.8 years), the frequency of CD4+ T cells was slightly reduced in preterms (GA <30 weeks) compared to controls (78).

#### 3.1.2 T-cell subsets

##### 3.1.2.1 CD4^+^ and CD8^+^ T-cell subsets

Although often both CD4^+^ and CD8^+^ T-cell subsets are reduced in number, this reduction seems to be most pronounced in CD4^+^ subsets, leading to an increased CD8^+^/CD4^+^ ratio in preterm neonates (16, 56, 59, 61, 79–81). For example, lower numbers of total T cells and CD4^+^ T helper cells were reported at birth in infants born at <36 weeks gestation, whereas CD8^+^ cytotoxic T cells were only reduced in the most premature group (<32 weeks) (18). The relative abundance of CD8^+^ T cells may reflect their role in prematurity-related immune activation: for example, increased CD8^+^ T-cell infiltration has been found in fetal placental membranes from pregnancies complicated by spontaneous preterm birth or intrauterine infection (80).

##### 3.1.2.2 Naive versus memory T-cell subsets

The fetal and neonatal T-cell compartments predominantly consist of naïve CD45RA^+^ T cells (82). Upon antigenic stimulation, naïve T cells upregulate tissue-specific homing receptors (e.g. α4β7 for migration to the intestines and CCR4 for non-gastrointestinal sites) (83), and differentiate into effector cells and memory cells expressing CD45RO. Both preterm and term neonates have high frequencies of naïve T cells and low frequencies of memory T cells compared to adults (15, 81, 84, 85). However, the literature is contradictory regarding the distribution of naïve, activated, effector, and memory T cells in preterm and term infants. Several studies, mostly using cord blood or peripheral blood samples collected within the first week of life, report a comparable distribution between preterm and term neonates (5, 15, 57, 82, 86, 87). In contrast, others describe a relative reduction in naïve CD4^+^ T cells (81, 88), naïve regulatory T cells (Tregs) (89), or both naïve CD4^+^ and CD8^+^ cells (59), accompanied with an increased proportion of CD8^+^ and CD4^+^ effector or central memory cells in preterm infants compared with controls (17, 80, 90).

The increased proportion of T cells expressing CD45RO and other activation markers reported in some studies may result from intrauterine and perinatal exposures that drive T-cell activation and proliferation. For example, Crespo et al. found a higher frequency of CD45RO^+^ T cells in preterm cord blood, particularly in infants exposed to chorioamnionitis, suggesting that intrauterine inflammation may drive premature T-cell activation, differentiation and proliferation (91). This aligns with findings from a recent study showing that activated central memory T cells are more abundant, whereas naïve T cells are less abundant in preterm infants exposed to histologic chorioamnionitis (92). Other studies have supported this concept of T-cell activation with the finding of increased CD25 (a marker of T-cell activation) expression in preterm newborns (85, 90, 93). Consequently, the variability in observed subset composition across the literature may be largely attributed to differences in study population. While some studies excluded only infants with clinical signs of chorioamnionitis (17), others excluded both cases with histological or clinical chorioamnionitis (15), and others do not mention how infection was handled (80). In the days after birth, T-cell subset composition continues to change. One study reported an absolute increase in naïve CD4^+^ T cells in preterm infants during hospitalization, accompanied by a relative decrease in antigen-experienced memory T cells (55). Another study found that the proportion of CD45RO^+^ T cells increased in the first week of life in both preterm and term neonates, but the increase seemed less pronounced in the preterm infants, potentially reflecting a delayed maturation or antigen exposure response in the preterm infants (84). Together, these findings suggest that the higher memory T-cell frequencies found at birth in some preterm neonates result from perinatal T-cell activation and subsequently decline as ongoing postnatal thymic output causes the naïve T-cell pool to expand.

##### 3.1.2.3 T helper cell subsets

CD4^+^ T cells differentiate into several subsets upon stimulation, depending on the antigen and the cytokine environment. In neonates, there is a T helper 2 (Th2) bias marked by dominant IL-4 production while T helper 1 (Th1) function (e.g. IFN- γ production) is suppressed, accompanied by an increased frequency and suppressive activity of regulatory T cells (Tregs) (94–96). This immunological orientation provides an anti-inflammatory state, which is thought to be critical for maintaining maternal-fetal tolerance, facilitating commensal colonization, and preventing inappropriate immune activation during early life (97). T helper 17 (Th17) differentiation in neonates is easily activated in inflammatory conditions and thought to play an important role in protection from Gram-negative bacterial and fungal infections (98, 99).

In preterm infants, this Th2 bias is found to be even more pronounced with more Th2-associated soluble CD30 and undetectable levels of Th1-associated soluble CD223 (100), more IL-4, IL-13, and IL-10 production upon stimulation of CD4^+^ T cells (98) and higher expression of Th2-associated transcription factor GATA3 and lower Th1-associated transcription factor T-bet compared to term-born controls (86). Additionally, increased expression of genes promoting Th17 differentiation leads to increased Th17 frequencies and IL-17 production in preterm cord blood (60, 98, 101). Both preterm and full-term neonates exposed to histologic chorioamnionitis have higher Th17 frequencies compared to unexposed neonates (101). This finding suggest that the observed Th17 skewing is probably partly the result of intrauterine inflammation.

Treg cells are increased in preterm neonates compared with term controls, particularly in the most premature infants (5, 17, 19, 86, 101–108). Only one study reported no difference between preterm and term neonates (89). Most of these neonatal Tregs have a naïve phenotype and have a strong suppressive capacity (105, 109). In the study from Pagel et al., this capacity was even higher in preterm infants than in term controls, as reflected by a reduction in CD4^+^ T cell proliferation of 88% after adding Tregs from preterm infants, compared with a reduction of 39% after adding Tregs of term infants (105). The proportion of Tregs declines over time (102), and around 28 days of life, preterm infants no longer have increased Tregs compared to term infants (105). Elevated Treg frequencies support immune tolerance in early life but may impair effective pathogen clearance, thereby contributing to the increased infection susceptibility observed in preterm infants.

##### 3.1.2.4 γδ-T cells

γδ T cells represent a minor subset of T cells, accounting for fewer than 10% of T cells in adults, that possess both innate and adaptive immune properties (110, 111). Unlike αβ T cells, γδ T cells can recognize antigens without the need for peptide presentation within an MHC molecule, enabling them to respond rapidly. Some authors have proposed that IFN-y production by γδ T cells is relatively intact in neonates as compared to adults, and that these cells therefore play an important role in the perinatal period in the absence of a fully competent αβ-T cell compartment (112). Some studies report reduced frequencies of γδ T cells in preterm infants compared to term-born infants and adults (17, 113–115), whereas others describe increased proportions (116). In the latter study, the proportion of naïve cells within the γδ T compartment was reduced, and the proportion of effector and effector memory subsets was increased compared to term infants. Notably, infants with sepsis within the first 14 days of life have higher frequencies of effector γδ T cells and lower proportions of naïve cells than those without sepsis (116), underscoring their response to infection. Over time, the frequencies of γδ T cells declined between two and 36 weeks postmenstrual age. These findings suggest that γδ T cells are generally reduced in preterm infants, but increased in case of infection.

#### 3.1.3 T-cell function

##### 3.1.3.1 T-cell receptor

The functional characteristics of T cells, including receptor diversity and signaling, proliferative capacity, responsiveness to stimulation and cytokine production, are key determinants of their ability to respond to pathogens. T-cell activation begins with antigen recognition by the T-cell receptor (TCR). Several studies have shown that the TCR repertoire in preterm infants shows developmental immaturity, including reduced gene diversity and shorter complementarity-determining region (CDR), mainly due to reduced insertion of nontemplated (N)-nucleotides (117). The CDR3 region is the most variable segment of the TCR and primarily responsible for peptide binding within the MHC molecule (118). Although TCR gene usage seems no longer limited by 24 weeks of gestation, the expansion pattern remains immature with an oligoclonal expansion up to 33 weeks of gestation, declining toward term (119). A less diverse TCR repertoire has been described up to 34 weeks of gestation (120, 121). This indicates that although the TCR recombination potential is present relatively early in gestation, the diversity continues to mature (117). In addition, the CDR3 length continues to increase with GA, and near-term neonates still have slightly shorter CDR3 regions than adults (119). Reduced TCR diversity and shorter CDR3 regions may affect antigen recognition and binding capacity, leading to lower affinity and specificity within the premature T-cell repertoire (122, 123). This functional TCR immaturity is supported by findings from a study where expression of genes involved in TCR signaling was lowest in extremely preterm infants and gradually increased with GA (124). Later, by 2–3 months after birth, preterm infants have TCR clonotype numbers and repertoire diversity comparable to term infants of equivalent postmenstrual age (125). Together, these findings suggest that although the TCR repertoire in preterms is initially developmentally immature, rapid postnatal diversification occurs.

##### 3.1.3.2 Proliferation

Early studies demonstrated that lymphocytes from preterm infants show a high rate of spontaneous DNA synthesis and proliferation, as well as increased apoptosis (126–128). This elevated turnover gradually declines over the first 4 to 9 weeks of postnatal life (126). A paper from 2003 confirmed that T-cell proliferation decreases during the last trimester, indicating that expansion of the peripheral T-cell pool mainly occurs early in the third trimester (128). Although lymphocytes of preterm infants show a higher baseline proliferation rate, most studies report reduced responsiveness to T-cell activators such as phytohemagglutinin (PHA) and concanavalin A (Con A), often proportional to GA (19, 129, 130). Interestingly, one study demonstrated that adult lymphocytes suspended in fetal plasma also showed reduced responsiveness, potentially due to high levels of immunosuppressive cytokines in fetal plasma (129). In addition, T-cell responsiveness to pathogen-associated antigens seems reduced as indicated by decreased proliferation upon stimulation with staphylococcal enterotoxin B (131) or with *Bordetella pertussis*, hepatitis B surface antigen (HBsAg), and *Haemophilus influenzae type b* after vaccination (16). Another report described reduced T-cell proliferation in response to influenza vaccination in children aged 6 to 18 months who were born preterm compared to healthy controls (132).

##### 3.1.3.3 Cytokines and immune regulators involved in T-cell function

Cytokine production is a central component of T-cell function. Compared to adults, neonates produce lower levels of pro-inflammatory cytokines (81, 115, 133), contributing to less effective responses to pathogens. In preterm infants, the reduced production of pro-inflammatory cytokines is even more pronounced as the increase in IFN-γ and TNF-α in response to bacterial, viral, or mitogen stimulation is often diminished (100, 102, 112, 115, 131, 134, 135). Several other pro-inflammatory cytokines are also reduced in preterm neonates, including IL-6, IL1β and IL-17 (17). However, functional maturation occurs with age (136). In one study, IFN-γ production by CD4+ and CD8+ T cells after *in vitro* stimulation with tetanus toxoid was comparable between preterm and term-born children before (15 months of age) and after (18 months of age) vaccination, suggesting that somewhere in the first year of life, T cells acquire their full potential of IFN-γ production (137). Other studies support this notion of maturation, with negligible IFN-γ production upon stimulation with staphylococcal enterotoxin B in both groups during infancy, but detectable production emerging around 12 months of age (74), and no significant differences in cytokine responses by the age of three (52).

In addition to reduced pro-inflammatory cytokines, IL-7, a cytokine important for T- and B-cell development and survival in the bone marrow, is also lower in preterm infants, along with reduced IL-7 receptor expression (5). The expression of the IL-7 receptor was positively correlated with the number of recent thymic emigrants (T-cell precursors) and total T-cell counts, stressing the role of IL-7 in T-cell development.

On the other hand, the production of several other cytokines seems to be relatively preserved in preterm infants. Several studies report that IL-2 levels in preterm and term-born neonates are equal to or even higher than those in adults (82, 134, 138, 139), suggesting that IL-2 production and IL-2 receptor expression are established early in fetal development. In one study, the proportion of IL-2 producing cells was inversely related to GA at birth, with differences no longer present at 12 months of age (74). Similar findings were reported for IL-4 (131, 140), IL-5 and IL-13 (102), with IL-5 levels being even higher in preterm compared to term born infants. These findings align with the described Th2 bias and immune suppression in early life. However, findings are not entirely consistent. One report described reduced levels of many cytokines, including IL-2 and IL-13, in preterm neonates (49). A longitudinal study in preterm infants with a mean GA of 33 weeks found that serum concentrations of IL-4 and TGF-β increased from birth to day 14, followed by a decline by day 28 (141). Together, these findings underscore the complex nature of cytokine regulation in early life. In particular, the reduced production of pro-inflammatory cytokines upon stimulation may contribute to a suboptimal immune response during pathogen invasion. This functional impairment is illustrated in an experiment showing reduced inhibition of *Candida albicans* by lymphocytes from both term and preterm neonates compared to lymphocytes from adults (142).

Additional mechanisms may contribute to reduced T-cell function in neonates. For example, a study showed that lysosomal activity in lymphocytes increases with GA toward term. Children aged 7–14 years had the highest levels, followed by a gradual decline through adulthood into older age (91). Lysosomes are important for T-cell function, including signaling, cytotoxicity and protein degradation (143). Moreover, membranes of preterm lymphocytes contain fewer arachidonic and docosahexaenoic acid, fatty acids that are important for the production of molecules involved in immune function such as prostaglandins and leukotrienes (59).

### 3.2 B cells and humoral immunity

#### 3.2.1 Maternally-derived antibodies

At birth, most circulating IgG is maternally derived. Since transplacental transfer of IgG by the neonatal Fc receptor (FcRn) mainly occurs in the third trimester (144, 145), preterm infants have significantly lower total IgG levels than term infants (146–153). For example, infants born at 25–28 weeks gestation have a mean serum IgG concentration of 251 (95% CI 114-552) mg/dL (154), compared to 953 (95% CI 855-1051) mg/dL in term infants (155). As a result, levels of maternally derived IgG against various pathogens and vaccine antigens are significantly lower as well, especially in those born before 30–32 weeks of gestation, irrespective of birth weight (145, 149, 153, 156–170). This deficit places preterm infants at increased risk for infection.

Interestingly, despite the overall lower antibody levels in preterm infants, functional antibodies with high affinity and avidity are present, suggesting a degree of selective transfer that may help partially overcome low concentrations (170, 171). The prophylactic use of intravenous immunoglobulin (IVIG) has been investigated as a potential strategy to enhance humoral immunity and reduce infection risk in preterm neonates. A study from 1994 demonstrated that a single dose of IVIG may not be effective in preventing late-onset sepsis in preterm infants (172). However, a more recent review from 2020 reported a minimal beneficial effect, showing a slight reduction in the incidence of late-onset sepsis but no significant decrease in mortality or other major morbidities (173). These findings suggest that, while IVIG may minimally influence immune defense mechanisms, its clinical application remains limited.

#### 3.2.2 B-cell numbers

B-cell development starts in the fetal liver by 7 weeks of gestation and later predominantly occurs in the bone marrow, as extensively described elsewhere (174–176). In contrast to the clearly reduced T-cell numbers, findings on total B-cell numbers in preterm infants are less consistent. In general, B-cell numbers increase during the early postnatal period, reaching high levels in infancy, and thereafter gradually decline throughout childhood and adulthood (177). Several studies report lower absolute B-cell numbers during the first weeks of life in preterm compared to term infants (5, 18, 55–57), with the lowest numbers in very preterm neonates (GA <32 weeks) (57, 58). For example, median B-cell counts in cord blood of preterm infants were 518 cells/µL (25^th^-75^th^ percentile, 348-804) versus 746 cells/µL (554–1056) in term infants. Similarly, at 6–8 weeks of age, preterm infants had a median of 931 B cells/µL (5^th^-95^th^ percentile, 466-2327), compared to 1481 B cells/µL (776–2358) in term infants (5, 56). In line with this, preterm infants often show lower levels of Kappa-deleting Recombination Excision Circle (KREC) in newborn screening assays (63, 66, 70, 73). KRECs are DNA fragments generated during B-cell receptor (BCR) formation and are therefore used as a marker for newly formed B cells. Their levels increase with GA, especially until 20 weeks of gestation (117). After birth, B-cell numbers increase in the first weeks to months (18, 88, 136), and seem to reach levels comparable to term infants somewhere between 2 and 7 months (55, 56). However, other studies did not observe significant differences in B-cell counts (15, 59, 88, 178) or KREC levels (62, 69) between preterm and term infants. Notably, most of the studies that did not find differences in B-cell numbers included only small numbers of term-born controls. Overall, although findings are not entirely consistent, the available evidence points toward reduced B-cell numbers in preterm infants, particularly those born at the lowest GA (**Figure 1**).

#### 3.2.3 B-cell subsets

Studies describing B-cell subsets in preterm infants are limited. Across all neonatal groups, the B-cell population consists of a higher proportion of naïve cells and a lower proportion of memory cells compared with adults, but no differences between preterm and term-born infants have been observed (15). In preterm fetuses (GA 17–28 weeks), the proportion of immature (CD10^+^) B cells in cord blood is higher compared with term neonates (179). Similar trends have been observed for infants born at <32 weeks gestation compared to infants born at 33–36 weeks or at term, although these differences did not reach statistical significance (88). Because immature B cells have limited capacity to respond to pathogen, a relative predominance of these cells may reduce the effectiveness of pathogen-specific immune responses. Transitional B cells (CD24^+^CD38^+^), representing peripheral immature B cells, were more abundant in preterm infants in one study (17), while another found no difference (140). The latter study found alterations in the regulatory B-cell population; both the frequency and the ability of B cells to differentiate into regulatory B cells were reduced in preterm infants (140). This, in combination with a pro-inflammatory cytokine profile in the context of infection, may contribute to impaired immune regulation. Furthermore, the proportion of CD11α-lacking B cells is higher in preterm infants, particularly those born at lower gestational ages (87). As CD11α is required for precursor cells to develop into mature B cells (180) and plays a role in interaction between B cells (181), its absence may be associated with the reduced proportion of mature B cells. In one study, B cells from preterm infants showed reduced expression of CD23 and CD21 (182), which are both involved in several B-cell functions such as complement binding and proliferation (183). Studies assessing B-cell development in preterm compared with term infants beyond the neonatal period are absent. Unpublished preliminary data from our group indicate that an immature B-cell subset distribution persists at 1 year of age in preterm-born infants. Taken together, although studies are scarce, evidence suggests that preterm neonates have alterations in B-cell subsets that reflect immaturity and may contribute to impaired immunocompetence.

#### 3.2.4 B-cell function

##### 3.2.4.1 B-cell receptor

BCR diversity increases with GA. Diverse gene usage in the Ig heavy chain is limited in early gestation (12–14 weeks), but shifts to a polyclonal pattern from week 17 onwards, and by week 22-26, this is comparable to the repertoire from healthy children (aged 9 months to 4 years) (117). However, others report repertoire alterations in preterm neonates, such as an overrepresentation of certain gene segments (i.e., D_H_7-27) in the diversity region of the heavy chain after 25–30 weeks gestation (184–186), as well as an increased occurrence of similar gene segments across multiple individuals (public clones) in preterm infants with a mean GA of 34 weeks (121). This indicates that although the gene usage is no longer limited in the third trimester, additional diversification still takes place. Furthermore, preterm infants have shorter CDR3 regions (117, 121, 184–187). The CDR3 region is the most variable segment of the BCR and primarily responsible for antigen binding (188). A reduction in length significantly decreases antigen-binding diversity, since each randomly inserted N-nucleotide can increase diversity by 20-fold (51). After preterm birth, CDR3 lengthening is not accelerated, but increases at the same speed as during the last trimester (186). From week 40 to 50 of postconceptional age, CDR3 length is similar in preterm and term infants and steadily increases during the first months of life independent of antigen exposure (185–187). In addition, although somatic hyper mutations (SHMs) are rare in all neonates, preterm infants show fewer SHMs in their Ig variable region than term infants and the postnatal rate of mutation accumulation is comparable between the two groups (184, 186). The reduced diversity, shorter CDR3 regions and fewer mutations contribute to a polyreactive binding pattern with lower BCR affinity in preterm neonates (189, 190). However, studies on how these developmental constraints affect plasma cell and memory B-cell response to infection or vaccination remain scarce.

##### 3.2.4.2 Antibody production

Several studies indicate that preterm neonates have impaired antibody production, with infants born at 30–36 weeks showing weaker IgG responses to cow milk proteins than term infants, largely normalizing by 6 months (191, 192). In another study, preterm infants with an infection secreted higher levels of IgG during the first postnatal week but reduced responses to subsequent *in vitro* stimulation with *E. coli* at a later time point. The authors suggested that “activation of ‘immature’ B-cells by pathogens could lead to exhaustion of Ig secretion*”* (193). Furthermore, the induction of IgG production via CD40 stimulation and the expression of several other B-cell activation receptors (i.e. BAFF-R, TACI and BCMA) were lower in preterm versus term cord blood or adult controls (194).

Fetal antibody production is low and predominantly of the IgM isotype (195). In neonates with intrauterine exposure to malaria in Cameroon, IgM concentration against *Plasmodium falciparum* was comparable between term and preterm neonates (196). Moreover, a similar proportion of infants produced IgM to all five tested *P. falciparum* antigens, suggesting no major restriction in antigen-specific B-cell responsiveness. Class-switch recombination occurs from around week 22 (117), and IgA transcripts can be detected from week 27 (185). Nevertheless, both term and preterm neonates have very low serum IgA at birth (197). In mucosal compartments, IgA levels are approximately 2.5-fold higher in term compared to preterm infants (198). Although the difference did not reach statistical significance, preterm neonates tended to show weaker intensity of reactive bands in their IgA responses to *Streptococcus mitis* and *Streptococcus mutans*, suggesting a less diverse antibody response (198).

### 3.3 Response to vaccination

The reduced capacity of preterm infants to produce antibodies can be further explored by examining their responses to vaccines administered in early life. Vaccination is arguably one of the most effective public health interventions, having averted an estimated 154 million deaths globally since 1974 (199). Since preterm infants are especially vulnerable to infections, it is even more important to protect them against vaccine-preventable diseases. While vaccine efficacy has rarely been assessed in preterm infants because of the large sample size required, their safety and immunogenicity have been described in more detail elsewhere (200–203). Here, we summarize the most important findings in the context of the developing preterm immune system.

#### 3.3.1 Safety

Although studies directly comparing term and preterm infants are scarce, systematic reviews describe similar systemic and local reactogenicity in preterm versus term infants, and a rare occurrence of serious adverse events (201, 204). Some studies found reduced injection-site reactions in preterms (205), raising the hypothesis that this may result from an impaired immune response. Cardiorespiratory events such as apnea, bradycardia, and desaturation can occur in preterm infants (206). These can mostly be managed with minimal intervention and appear primarily associated with pre-existing clinical cardiorespiratory instability.

#### 3.3.2 Immunogenicity

The tetanus and diphtheria toxoid antigens used for vaccination are highly immunogenic, and a relatively low antibody titer (i.e. ≥0.1 IU/mL) is sufficient for protection. As a consequence, the majority of preterm infants achieve protective levels of diphtheria- and tetanus-specific IgG, comparable to term infants (207–209), also when administered as a hexavalent diphtheria-tetanus-acellular pertussis-inactivated poliovirus-*Haemophilus* influenzae type b-hepatitis B (DTaP-IPV-Hib-HepB vaccine (210–212).

Preterm infants also almost always reach seroprotective antibody levels after a primary series of vaccination with inactivated polio vaccine, nowadays most often included in a pentavalent or hexavalent formulation (210, 213–215). Although some studies show lower titers in preterm infants (213, 214), these are above protective thresholds and persist into childhood after booster doses (216).

Acellular pertussis-containing vaccines are immunogenic and effective in preterm infants (210, 211, 213, 214), but they elicit lower antibody concentrations than in term-born infants (211, 217). There is no established correlate of protection for pertussis, but increased breakthrough disease rate in preterm infants supports the notion of reduced vaccine effectiveness (218, 219). Vaccine effectiveness for pertussis was estimated by one study at 73% versus 95% for the first dose and 86% versus 99% for the primary vaccination series in preterms versus terms, respectively (220).

Preterm infants consistently mount measurable but lower Hib antibody responses compared with term infants after the primary series (16, 209, 211, 212, 221), with 41% reaching levels above the threshold of 0.15ug/ml after the primary series, versus 84% in term-born infants (211). Booster doses largely reduce this gap (211, 222), and most children, including preterms, retain protective levels into school age (223, 224).

For hepatitis B immunization, the data are variable. While some studies show lower titers and seroconversion rates in preterm infants (16, 225–227), others report seroprotection rates and antibody titers comparable to term-born infants (212, 223, 228). Because of the reported reduced immunogenicity of hepatitis B vaccination in infants weighing <2000 g, the American Academy of Pediatrics advises delaying the first dose for a month or until hospital discharge for infants with hepatitis B surface antigen-negative mothers (229).

Data on rotavirus vaccination of preterm-born infants are relatively limited, but suggest good immunogenicity and substantial protection (230, 231).

The WHO recommends vaccination against measles, mumps, and rubella (MMR) at 9 months in countries with high measles incidence and mortality; in countries with a low incidence, the first dose is delayed until 12–15 months of age. In one study, vaccination with the MMR combination vaccine at 12 months resulted in protective antibody levels against measles in 100% of preterm infants with a birth weight of <1500 g, with titers similar to those of term-born infants (232). Another study in children born <29 weeks of gestation showed no difference in antibody titers with term infants for all three viruses, and 100% seroprotection for measles (233). Persistence of measles antibodies was demonstrated in all preterm- and term-born children at 5–6 years of age (224). The proportion of preterm children with protective antibodies against mumps and rubella was lower, 76 and 80%, respectively. This did not differ from term-born children and is in line with the reported faster waning of rubella and mumps antibodies, compared to measles (234).

Vaccination with the 10-valent pneumococcal conjugate vaccine resulted in lower serotype-specific IgG concentrations in preterm infants compared to terms, but almost all children achieved levels above the established protective threshold of 0.35 ug/ml after a booster dose at 11 months (211). Similar results were found using the 13-valent pneumococcal conjugate vaccine (235).

In summary, the ability of preterm infants to mount robust antibody responses after vaccination depends on the antigen and vaccine composition, with some vaccines yielding lower antibody titers but generally protective levels. Therefore, despite preterm infants being immunologically less mature, the general advice is to administer vaccines chronologically, without correction for gestational age, to protect them early in life (229, 236).

## 4 The effect of infection on the preterm immune system

As noted earlier, not only GA but also exposure to antenatal and postnatal infection affects immune development in preterm infants. Several studies have reported an activated immune status at birth in preterm infants exposed to chorioamnionitis, including increased proportions of memory T cells (91), higher expression of activation markers such as CD69 and CD35 (136), elevated Th17-associated gene expression, increased IFN-γ expression (237), increased proportions of monocytes (238), and elevated levels of pro-inflammatory cytokines such as IL-8 and IL-6 (239, 240), accompanied by reduced suppressive activity of Tregs (240) as compared with preterm infants without infection. In one study, elevated inflammation-related proteins at birth, including TNFα, IL-8, and IL-6, remained elevated at day 7, even after adjustment for presumed or definite bacteremia (241). At the same time, monocytes from preterm infants exposed to chorioamnionitis show a hyporesponsive transcriptional profile after stimulation with *S. epidermidis*, with reduced expression of a subset of genes involved in antigen presentation and adaptive immune activation compared with those from unexposed preterm infants (238). Similarly, poorer IL-8 responses during the first three months of life were reported in preterm infants exposed to chorioamnionitis than in unexposed preterm infants (136), supporting a hypothesis of immune exhaustion, although IL-2 and TNF-α production were not impaired. Notably, GA may have contributed to these findings, as infants in the stable, unexposed cohort were born at a later GA.

The impact of prenatal infection on the immune system is relevant, as infants born in the setting of chorioamnionitis also had higher rates of microbiologically confirmed sepsis and chronic lung disease than the stable, unexposed cohort (136). The association between intrauterine infection and subsequent immune-related morbidities such as BPD has been reviewed elsewhere (242, 243). Importantly, these authors emphasize that substantial confounding by GA and other clinical factors makes it difficult to disentangle the underlying causal mechanisms.

On the other hand, altered immune function may precede the onset of neonatal infection. In one study, preterm infants who later developed late-onset sepsis (median onset 13 days) produced significantly lower levels of most measured cytokines and chemokines, including IL-1β, IL-6, IL-8, TNF-α, MIP-1α, and MCP-1, before sepsis diagnosis. This decrease became more pronounced after the sepsis episode, consistent with the theory of transient immune paralysis after sepsis (244). Although the authors adjusted for relevant confounders, including GA, prenatal infection was not accounted for, which is relevant given the association between chorioamnionitis and neonatal sepsis (245). In another study, preterm infants who later developed late-onset sepsis had lower absolute neutrophil counts on day 1 (246). Together, these data suggest that while preterm immunity is characterized by immaturity, preterm birth in the context of antenatal or postnatal infection is associated with early immune activation that may be followed by functional hypo-responsiveness or exhaustion. However, most available studies are cross-sectional, include small or heterogeneous cohorts, and do not fully disentangle the effects of GA, intrauterine inflammation, and neonatal infection. Longitudinal studies that stratify preterm infants according to antenatal and postnatal infection exposure will therefore be essential to separate the effects of infection from those of prematurity itself on immune system development.

## 5 Conclusion and future perspectives

Preterm-born infants show immaturity in both innate and adaptive immune responses— including reduced cell numbers, altered subset composition, and impaired effector functions—that is inversely proportional to GA. This developmental immaturity contributes to the increased risk of infection and infection-related morbidity and mortality in this population. Future longitudinal studies that stratify preterm infants by antenatal and postnatal exposures, such as infection, are essential to separate the effect of external factors from prematurity itself on immune development.

While this review has focused on the current understanding of the immune system development in preterm infants, identifying strategies to improve immune cell function is an important area for future research. Several interventions have been explored, including administration of complement factors (247), IVIG (173), granulocyte-macrophage colony stimulating factor (GM-CSF) (248), or colostrum (249, 250). While some studies report small improvements in specific immune measures, larger trials and meta-analyses have generally failed to demonstrate reductions in major outcomes such as mortality. Probiotic supplementation remains one of the more promising options, with evidence for reducing necrotizing enterocolitis and some infection outcomes (251, 252), although the benefit for the most premature infants with a birth weight <1000g remains questioned.

Immune immaturity in preterm infants involves many components of the immune system. Recognizing this all-encompassing nature of immune immaturity is important for both clinicians and researchers, as it guides clinical practice and research toward integrated, multi-targeted strategies to improve health outcomes.
